# PP88 Life Cycle Scientific Advice For Health Technology Assessment In Korea

**DOI:** 10.1017/S026646232510305X

**Published:** 2025-12-29

**Authors:** Jihyun Kang, Yujin Kang, Wonjung Choi, Jieun Choi

## Abstract

**Introduction:**

Scientific advice (SA) in the process of developing medical devices and technologies (MedTechs) allows manufacturers to target the profile of their production and gain a better understanding of the requirements for approval and reimbursement. The aim of this study was to evaluate the SA framework developed by National Evidence-based Healthcare Collaborating Agency (NECA) in Korea.

**Methods:**

We developed the SA program by reviewing all available information regarding the service offered, processes, duration (in weeks), stage of development, types of technologies, and fees. The SA program supports a total of 60 MedTechs per year. By the end of 2023, a total of 150 technologies had participated. We also evaluated participant satisfaction with the SA program after their participation.

**Results:**

The NECA SA program offers Evidence Preview, Evidence Search Education, Evidence Development Plans with regulatory advice, and Clinical Expert Advice for clinical trial design. The process is flexible, depending on the applicant’s needs, and is completed within 24 weeks, with a final report issued after completion. Stage of development and type of technology are mainly focused on early stages of development (110/150 technologies) and innovative technologies (87/150 technologies). Participant satisfaction with the SA program was more than 90 percent for the past three years. The SAs offered by NCEA are optional, free of charge, confidential, and non-binding.

**Conclusions:**

This framework helps manufacturers understand the evidence requirements for NECA and other payers. Considering that most MedTechs are in the early stages of development, it is important to develop a target technology profile (TTP) tool to prepare guidelines for MedTech development plans. In addition, there is growing international collaboration between industry and regulatory authorities. It may also be beneficial to engage with specialized consulting institutions.

